# Oxidative stress enhanced the transforming growth factor-β2-induced epithelial-mesenchymal transition through chemokine ligand 1 on ARPE-19 cell

**DOI:** 10.1038/s41598-020-60785-x

**Published:** 2020-03-04

**Authors:** I-Hui Yang, Jong-Jer Lee, Pei-Chang Wu, Hsi-Kung Kuo, Yu-Hsia Kuo, Hsiu-Mei Huang

**Affiliations:** Department of Ophthalmology, Kaohsiung Chang Gung Memorial Hospital and Chang Gung University College of Medicine, Kaohsiung, Taiwan

**Keywords:** Stress signalling, Mechanisms of disease

## Abstract

Fibroblast-like transformation of retinal pigment epithelial (RPE) cells is a pathological feature of proliferative vitreoretinopathy (PVR) that may cause blindness. The effect of oxidative stress alone or together with transforming growth factor-beta 2 (TGF-β2) on epithelial-mesenchymal transformation (EMT) is not fully understood in RPE. TGF-β2 induced the upregulation EMT markers including α-smooth muscle actin (α-SMA), Snail, and Slug and downregulation of E-cadherin (E-cad) in ARPE-19 cells. Hydrogen peroxide (H_2_O_2_) not only upregulated α-SMA but also enhanced the effect of TGF-β2 on the expression of Snail and Slug. The CXCL family of cytokines could be the mediators of EMT induced by H_2_O_2_ and TGF-β2. H_2_O_2_ induced CXCL1, that upregulated α-SMA and fibronectin. Both SB225002, an inhibitor of CXCR2, and antioxidant N-acetylcysteine suppressed the TGF-β2-induced EMT in ARPE-19 cells. Taken together, the results suggest that oxidative stress enhanced TGF-β2-induced EMT through the possible autocrine effect of CXCL1 on CXCR2 in ARPE-19 cells.

## Introduction

The formation of fibrosis in tissues is a complication that follows the epithelial-mesenchymal transition (EMT) of the resident cell^1^. During the process of EMT, epithelial cells undergo a mesenchymal change, which leads to increased cell migration and the loss of cell-to-cell contact as well as the apical-basal polarity^2,3^. Oxidative stress plays a role in the molecular mechanism underlying fibrosis in a variety of organs, including the lung and liver^4–7^. Increased oxidative stress in cells and tissues has been implicated in the pathogenesis of retinal diseases threatening vision.

After a long-term disease, fibrosis and scaring are observed in the epiretinal or subretinal spaces. In a study involving lung fibrosis, Jain *et al*. showed that inhibition of transforming growth factor-beta (TGF-β)-induced reactive oxygen species (ROS) generation decreased the expression of pro-fibrotic genes, including α-smooth muscle actin (α-SMA) and connective tissue growth factor (CTGF). ROS generated by complex III of the electron transport chain was found to be essential for TGF-β-induced fibrosis in the lung^8^. The retina is highly susceptible to ROS-induced injuries due to its high rate of oxygen consumption, high exposure to light energy, and high polyunsaturated fatty acid content^9^. Many retinal diseases, including age-related macular degeneration (AMD) and retinal detachment (RD), have been demonstrated to be associated with excessive ROS formation^9–12^. Abnormal fibrosis of the retinal tissue could be associated with EMT of retinal pigment epithelial (RPE) cells in the pathological environment. It has been demonstrated that oxidative stress induces cell-cell dissociation, the initial step in EMT^13^. In addition, it has been reported that TGF-β alone may not be sufficient to initiate the loss of cell-cell contact, but plays a role in inducing transition of RPE cells to a myofibroblast phenotype, which is associated with fibrotic complications such as proliferative vitreoretinopathy (PVR)^14^.

Increase in ROS levels has been demonstrated to be associated with the regulation of expression of CXC chemokines in epithelial tumor cells^15^. Among the chemokines, interleukin-8 (IL-8, also known as CXCL8) secreted by tumor cells potentiates tumor progression by inducing adjacent epithelial tumor cells into EMT^16^. In the retina, the effect of ROS in the regulation of expression of CXC chemokines and their role in EMT of RPE cells is still not fully understood. The purpose of this study was to evaluate the effect of ROS in TGF-β2-induced EMT and the involvement of CXC chemokines in the regulation of EMT-associated genes in RPE cells.

## Results

### H_2_O_2_ and TGF-β2 upregulate the markers of EMT in ARPE-19 cells

**Figure 1:**
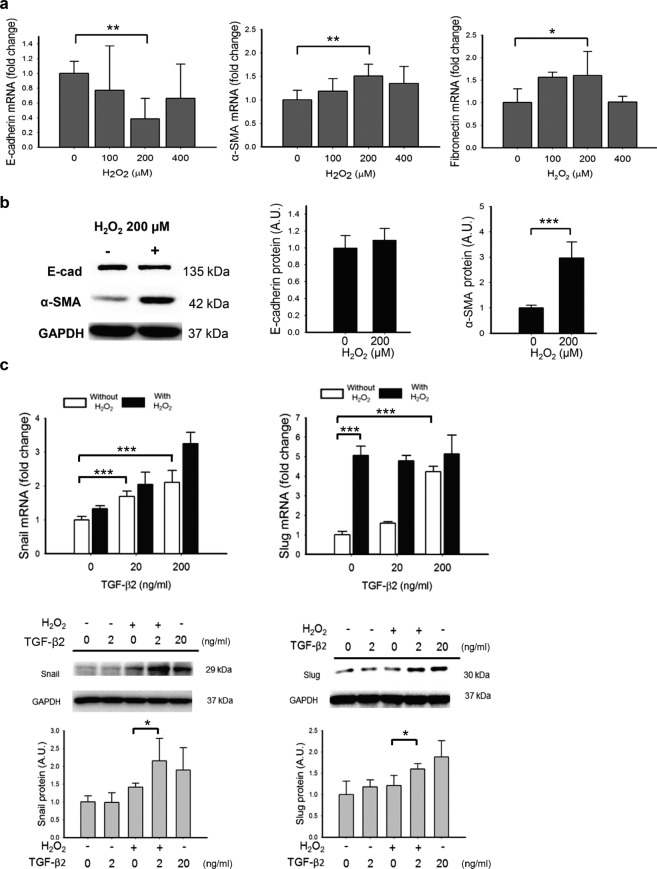
Hydrogen peroxide (H_2_O_2_) enhanced the TGF-β2-induced EMT in APRE-19 cells. **(a)** The changes in transcription of mRNA of EMT-associated markers: E-cad, α-SMA and FN was induced by 200 µM H_2_O_2_. **(b)** The H_2_O_2_ at 200 µM concentration induced an upregulation of α-SMA protein, but did not affect the protein expression of E-cad. **(c)** TGF-β2 upregulated the mRNA of transcription factor Snail and Slug in a dose-dependent manner in cells without (white bar) or with (black bar) exposure to H_2_O_2_. Slug mRNA was upregulated in cells treated with H_2_O_2_ (200 µM). Combined presence of H_2_O_2_ further enhanced the effect of TGF-β2 (20 ng/mL) on protein expression of Snail and Slug. (Results of 4 repeated experiments; *p < 0.05; **p < 0.01; ***p < 0.001).

To assess the effect of oxidative stress and TGF-β on EMT, ARPE-19 cell line was treated with H_2_O_2_ (100–400 µM) and/or recombinant human TGF-β2 for analyzing the expression of EMT markers including E-cad, α-SMA, and fibronectin (FN). Similar to the results of previous studies^17,18^, TGF-β2 induced a dose-dependent decrease in E-cad mRNA, an increase in FN mRNA, and a non-significant upregulation of α-SMA mRNA. Western blotting of whole-cell lysates of ARPE-19 was consistent and showed that TGF-β2 (20 ng/mL) decreased E-cad and increased α-SMA protein levels compared to ARPE-19 cells treated with DPBS (Supplementary Fig. S1). On the other hand, the cells treated with 100 and 200 μM H_2_O_2_ for 4 h exhibited lower levels of E-cad mRNA and higher levels of α-SMA mRNA and FN mRNA 24 h after the initiation of treatment; however, the effect was not observed with higher H_2_O_2_ concentration of 400 μM (Fig. 1a). At 24 h after the initiation of treatment, while H_2_O_2_ (200 μM) significantly increased the levels of α-SMA protein, it had no effect on E-cad protein level, (Fig. 1b). High dose of TGF-β2 upregulated mRNA and protein expression of Snail and Slug, two important transcription factors associated with EMT. The presence of H_2_O_2_ at 200 μM significantly enhanced the effect of low-dose TGF-β2 on the expression of Snail and Slug at both mRNA and protein levels (Fig. 1c). Treatment of H_2_O_2_ for 4 h at a concentration below 400 µM or TGF-β2 of 20 to 40 ng/mL for 24 h did not change the cell viability when compared with PBS-treated ARPE-19 control cells (Supplementary Fig. S2).

### H_2_O_2_ and TGF-β2 induces C-X-C chemokine family and their receptors

**Figure 2:**
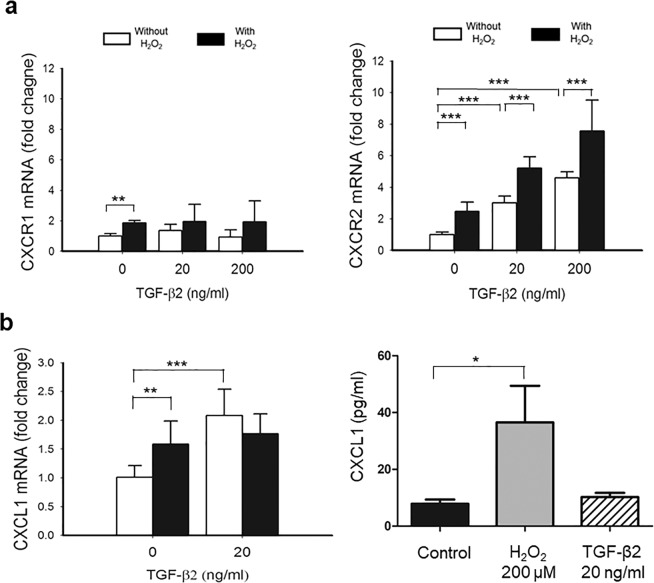
TGF-β2 induced C-X-C Receptor (CXCR) expression that was enhanced by H_2_O_2_ in ARPE-19 cells. **(a)** Treatment of TGF-β2 showed a dose-dependent upregulation of CXCR2 mRNA that was also significantly enhanced when cells were pre-exposed to 200 µM H_2_O_2_. A minor change in expression of CXCR1 mRNA in cells was also observed after the exposure to H_2_O_2._ **(b)** ARPE-19 cells treated with 200 μM H_2_O_2_ or 20 ng/mL TGF-β2 significantly upregulated CXCL1 mRNA than cells treated with DPBS as control. ELISA showed the expression of CXCL1 protein increased after treatment of ARPE-19 with sublethal concentration of H_2_O_2_ (200 µM, 4 h). (Results of 4 repeated experiments; *p < 0.05; **p < 0.01; ***p < 0.001).

Real-time qPCR was used to investigate the effect of TGF-β2 and H_2_O_2_ on the expression of both the ligands and receptors of C-X-C chemokine family. The levels of two types of receptors, CXCR1 and CXCR2, were detected with qPCR. The mRNA expression of both CXCR1 and CXCR2 were upregulated by 1.9 and 2.5 folds after treatment of H_2_O_2_ at 200 μM (Fig. 2a). After incubation in media containing TGF-β2, the expression of CXCR2 mRNA increased dose-dependently but no effect was detected on CXCR1. The TGF-β2-induced upregulation of CXCR2 was further enhanced in the presence of 200 μM H_2_O_2_ (Fig. 2a). In addition, ARPE-19 cells treated with 200 μM H_2_O_2_ showed a significant upregulation of CXCL1, CXCL2, CXCL3, CXCL6, and CXCL8 (IL-8) mRNAs than cells treated with DPBS as control (Fig. 2b, Supplementary Fig. S3). The upregulation CXCL1 in culture media of ARPE-19 after the treatment of H2O2 at sublethal level (200 µM, 4 h) was also confirmed with ELISA (Fig. 2b). The 20 ng/mL TGF-β2 treatment upregulated CXCL1, CXCL5, CXCL6, and CXCL8 and downregulated CXCL2 mRNA (Fig. 2b, Supplementary Fig. S3). The expression of CXCL5 was not detected in ARPE-19 cells treated with TGF-β2 at a concentration lower than 10 ng/mL or in the cells treated with H_2_O_2_ (Supplementary Fig. S3).

### CXCR2 inhibitor inhibits the expression of EMT-associated genes

**Figure 3:**
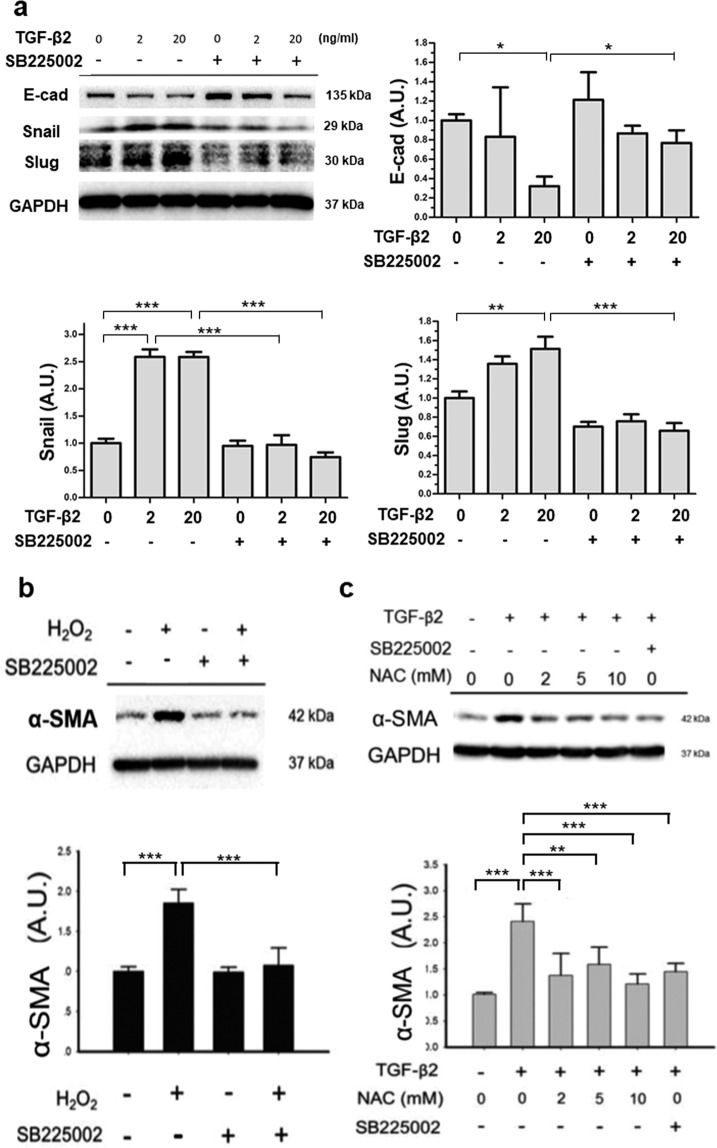
Inhibition of CXCR2 with SB225002 and anti-oxidant suppressed the change of EMT markers induced by TGF-β2 and H_2_O_2_ in ARPE-19 cells. **(a)** SB225002 (1.0 µM) inhibited the TGF-β2-induced protein expression of Snail, Slug and E-cad. **(b)** The over-expression of α-SMA induced by 200 µM H_2_O_2_ was inhibited by 1.0 µM SB225002. **(c)** The TGF-β2 (20 ng/mL)-induced α-SMA protein expression was inhibited by both anti-oxidant N-acetyl-L-cysteine (NAC) and 1.0 µM SB225002. (Results of 3 repeated experiments; *p < 0.05; **p < 0.01; ***p < 0.001).

The CXCR2 inhibitor SB225002 was used to block the action of a CXCL cytokine in ARPE-19 cells. It was found that the changes in the intracellular protein expression of Snail, and Slug, after treatment with TGF-β2 for 24 h in ARPE-19 cells, was significantly inhibited by SB225002 (Fig. 3a). The reduced E-cad expression upon 20 ng/mL TGF-β2 treatment was rescued by SB225002 (Fig. 3a). Pre-treatment with SB225002 significantly suppressed the H_2_O_2_-induced upregulation of α-SMA (Fig. 3b). Furthermore, pre-treatment of ARPE-19 cells with N-acetyl-L-cysteine (NAC), a known antioxidant, showed a comparable effect as that exhibited by SB225002, for inhibition of α-SMA protein expression induced by TGF-β2 (20 ng/mL) (Fig. 3c). These results suggested that oxidative stress could play a role in TGF-β2 induced expression of α-SMA, and CXCL chemokines could mediate the effect of oxidative stress on α-SMA upregulation in ARPE-19 cells.

### CXCL1 induces the changes in EMT-associated markers expression

**Figure 4:**
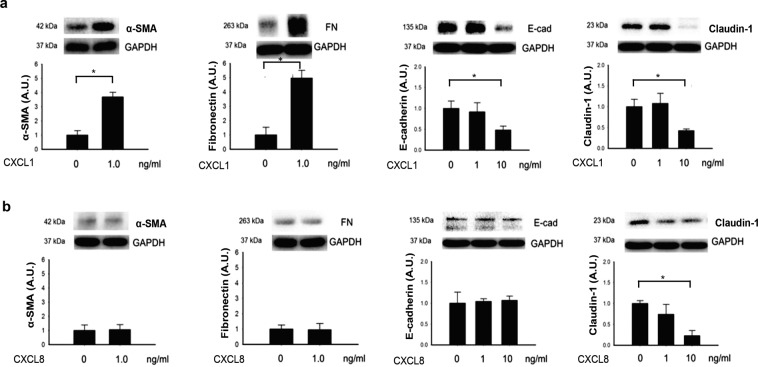
CXCL1 induced the changes of EMT-associated proteins. **(a)** Treatment with 1.0 ng/mL CXCL1 induced upregulation of α-SMA and FN. CXCL1 at 10 ng/mL concentration induced downregulation of E-cad and claudin-1 in ARPE-19 cells after 24 h. **(b)** Treatment of CXCL8 showed no effect on α-SMA, FN, and E-cad, but suppressed claudin-1 expression at 10 ng/mL concentration. (Results of 3 repeated experiments; *p < 0.05).

**Figure 5:**
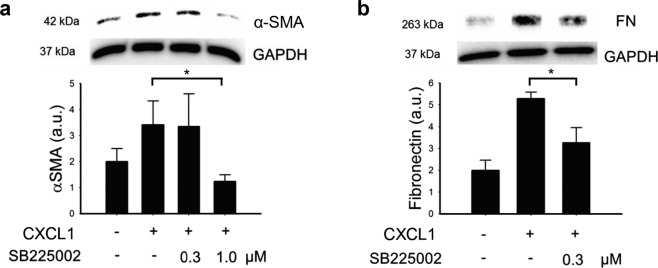
Inhibition of CXCR2 with SB225002 reverse the CXCL1-induced fibrotic proteins expression in ARPE-19 cells. The CXCL1-induced upregulation of protein expression of **(a)** α-SMA and **(b)** FN were suppressed with SB225002 at the concentration of 1.0 µM and 0.3 µM. (Results of 3 repeated experiments; *p < 0.05).

Treatment of APRE-19 cells with 1.0 ng/mL recombinant CXCL1 protein for 24 h increased the expression of α-SMA and FN proteins in the cells, when cultured in serum free medium supplemented with 20 ng/mL TGF-β2 (Fig. 4a), while, recombinant CXCL8 showed no significant effects in α-SMA and FN protein levels under the same experimental conditions (Fig. 4b). The degradation of epithelial markers, E-cad and claudin-1, was investigated. ARPE-19 cells treated with 10 ng/mL CXCL1 for 24 h showed a significant decrease in both E-cad and claudin-1 protein expression (Fig. 4a). CXCL8, up to a concentration of 10 ng/mL, showed no effect on E-cad but suppressed claudin-1 protein expression (Fig. 4b). Inhibition of the receptor of CXCR2 by SB225002 suppressed the CXCL1-induced upregulation in α-SMA and FN protein in cells treated with 20 ng/mL of TGF-β2 (Fig. 5). These results suggested that CXCL1 but not the CXCL8 facilitates the EMT change through CXCR2 in ARPE-19 cells.

## Discussion

**Figure 6:**
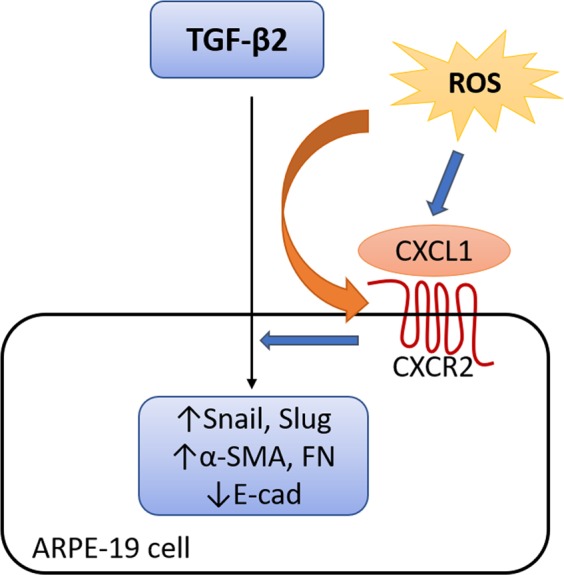
Diagram illustrating the main findings of this project. Both sub-toxic levels of H_2_O_2_ and TGF-β2 induce the upregulation of epithelial-mesenchymal transformation (EMT) associated transcriptional factors (Snail and Slug) and promote the expression of EMT markers (α-SMA↑, E-cad↓, FN↑). Synergistic effect of H_2_O_2_ and TGF-β2 augment the EMT via CXCL1 and CXCR2.

In this study, we demonstrated that low level of oxidative stress could potentially facilitate TGF-β2-induced EMT in ARPE-19 cells. At a sub-toxic level, H_2_O_2_ induced the mRNA expression of Slug, one of the important EMT-associated transcription factors. Both sub-toxic levels of H_2_O_2_ and TGF-β2 induce the expression of CXC chemokines and upregulation of CXCR2 in APRE-cells. The CXCR2 mainly responses to the CXC family of chemokine CXCL1 derived from RPE itself by possible autocrine regulation. A synergistic effect may exist between sub-toxic levels of H_2_O_2_ and TGF-β2 that contribute to the additional enhancement on the expression of Snail, Slug and CXCR2, resulting in the EMT in RPE cells (Fig. 6). Both CXCR2 inhibitor and anti-oxidant downregulated α-SMA. Therefore, the fibrosis of tissue in a variety of pathological diseases in retina including proliferative diabetic retinopathy (PDR), PVR, or other macular degenerative diseases could be associated to the synergistic effect of TGF-β2 and oxidative stress.

Both, the H_2_O_2_ and TGF-β2 at sub-toxic levels showed different effect on the expression of CXCL chemokines in ARPE-19 cells. H_2_O_2_ upregulated CXCL1, CXCL2, CXCL3, CXCL6, and CXCL8, whereas TGF-β2 upregulated CXCL1, CXCL5, CXCL6, and CXCL8. Both the types of chemokine receptors, CXCR1 and CXCR2, were expressed in ARPE-19 cells. Treatment of cells with TGF-β2 showed a dose-dependent effect in the expression of CXCR2 mRNA. We found that the blockage of CXCR2 with its specific inhibitor suppressed both the H_2_O_2_- and TGF-β2-induced α-SMA expression. Therefore, an autocrine effect of CXCL chemokines may explain an interactive effect between H_2_O_2_ and TGF-β on the EMT of RPE cells.

The effect of inflammatory cytokines on EMT and fibrosis has been reported. Shephard *et al*. showed that interleukin-1 (IL-1) inhibits TGFβ1-induced α-SMA expression in cultured dermal fibroblasts^19^. In contrary, recombinant IL-6 increases α-SMA transcription in fibroblasts derived from IL-6-null mice without exogenous TGF-β1^20^. CXCL chemokines have been reported to involve EMT of cancer cells and other disease models. CXCL1/GROα regulates NF-κB activation through the AKT pathway to increase cell migration and invasion in PC-3 and DU145 castration-resistant prostate cancer cell lines^21^. CXCL1 has also been demonstrated to play an important role in gastric cancer progression and cell migration^22^. In the retina, it has been demonstrated that CXCL1 levels correlated significantly with the duration of RD and PVR^23^. Increased CXCL1 levels may be indicative of mild inflammation in the detached retina and the adjacent vitreous^24^. In this study, sublethal dose of H_2_O_2_ alone upregulated CXCL1 protein in culture media of ARPE-19 (Fig. 2b). The exact signaling pathway through which ROS induced EMT is not clear. However not only in RPE, ROS has been demonstrated to induce EMT by activating Snail expression in SCp2 nontumorigenic mouse mammary epithelial cells and MCF-7 cells through pathway that was not directly correlated with TGF-β^25,26^.

The other types of CXCL chemokines may have distinct effects on EMT. The functions of CXCR2 may be multiple and vary depending on the cell type. Shen *et al*., demonstrated that CXCL8 protected human vascular endothelial cells from oxidative stress-induced senescence^27^. The expression of CXCL1 and CXCL8 is a cellular response to oxidative stress. CXCL8 or CXCR2 also had been reported to be associated with increased oxidative stress neutrophils and CD45+ cells in mouse aorta^28,29^. SB332235, another CXCR2 inhibitor, mitigated the amyloid-β-induced oxidative stress in rat brain^30^. CXCL1 was correlated with EMT, however, its effect on senescence of ARPE-19 cells is beyond the scope of this study. In a study on epithelial tumor, CXCL8 plays a role in the acquisition and/or maintenance of the mesenchymal and invasive features of tumor cells^16^. CXCL6 contributes to experimental pulmonary fibrosis induced with bleomycin in mice by PI3K/Akt/GSK-3β/Snail signaling^31^. Our finding showed that pharmacological inhibition of CXCL chemokines may have a role in inhibition of EMT and associated fibrosis in vitreoretinal diseases.

α-SMA is the actin isoform that predominates within vascular smooth-muscle cells^32^. In the kidney, α-SMA has been demonstrated to play an important role in fibrogenesis^7^. The expression of α-SMA is a characteristic of activated myofibroblast that synthesize the extracellular matrix proteins^33^. In a study on the skin, the incorporation of α-SMA into stress fibers increases contractile activity of sub-cutaneous fibroblastic cells and contributes to the contraction of connective tissue in tissue remodeling^34^. α-SMA upregulation by sub-toxic level of H_2_O_2_ in ARPE-19 cells indicates the possible role of oxidative stress in myofibroblastic change following the TGF-β-induced EMT of RPE cells. This finding suggests that low level of oxidative stress would worsen the hardening, fibrosis, and tractional detachment of retina in diseases associated with increased level of intravitreal or subretinal TGF-β.

In addition to α-SMA, CXCL1 also mediates the expression of FN. Increased FN expression in epithelial cells is one of the characteristics of EMT^3,35^. A three-dimensional fibrillar network of FN on the cell surface is critical for the tissue architecture and also plays a role in the migration and metastasis of cells^36–38^. The fibrillar network of FN has been shown to be required for proliferation and migration of human umbilical-vein endothelial cells in a three-dimensional environment^39^. During wound healing, FN has been demonstrated to be important in the migration of myofibroblasts and epithelial cells^40^. The presence of both alternatively spliced FN and TGF-β are required in the differentiation or trans-differentiation of cells to myofibroblasts which play a critical role in the process of tissue fibrosis^40,41^. In pathological conditions involving TGF-β and oxidative stress, excessive fibronectin production could be associated with the RPE migration and abnormal fibrovascular growth in vitreous or subretinal space.

Cadherin is the key component of the adherens junction and regulates cell–cell adhesion. For most epithelial cells, E-cad plays a role in assembly of cell–cell junctions and is the indicator of EMT^42^. Previous study reported N-cadherin, not E-cad, is the major component of cell-cell junction in APRE-19 cells. The oxidative stress triggers transient N-cadherin dislocation from the plasma membrane to cytoplasm causing disruption of cell–cell adhesion and stress fiber formation, but does not affect the expression of N-cadherin protein^13^. In this study, subtoxic H_2_O_2_ induced downregulation of E-cad mRNA, but did not affect the expression of E-cad protein. This inconsistency may be due to be downregulated E-cad mRNA that are not sufficient to decrease the protein expression by 4 hours after initiation of H_2_O_2_ treatment, or possibly because the E-cad may internalize from the cell border to accumulate in the cytoplasm. Further experiments will be done to confirm the expression and localization of cadherin at different time points *in vitro* and *in vivo* under oxidative stress.

In conclusion, either subtoxic TGF-β2 or oxidative stress is an inducer of EMT in RPE cells. Synergistic effect of oxidative stress and TGF-β2 augment the expression of EMT-associated transcriptional factors in RPE cells. CXCL1 is the main factor that mediates the oxidative stress- and TGF-β2- induced EMT through its receptor CXCR2. Pharmacological inhibition of CXCR2 suppresses EMT in RPE cells and could be a potential treatment for EMT-associated abnormal fibrosis in retinal diseases including PDR with tractional RD and PVR.

## Materials and Methods

### Culturing ARPE-19 cells

A total of 2.5 × 10^6^ ARPE-19 cells (ATCC, Manassas, VA) were seeded in a 60-mm dish (Corning, Tweksbury, MA) and cultured with Dulbecco’s Modified Engle’s Medium/F12 (Thermo Fisher, Waltham, MA) plus 10% of bovine serum albumin (Thermo Fisher) as well as 1% penicillin/streptomycin (Thermo Fisher) in a 37 °C humidified and 5% CO_2_ culture incubator. The cells were maintained in the 6 wells or harvested with trypsin (Thermo Fisher) and then seeded in 96 wells (Corning) before specific experiments.

### Treatments and reagents

At 80% subconfluent cell growth, the medium was replaced by serum free DMEM/F12 for 4 h prior to treatment. TGF-β2, CXCL1, or CXCL8 (PeproTech, Rock Hill, NJ) were added to the culture medium for 24 h in the individual experiments. The cells were incubated with 200 µM H_2_O_2_ for 4 h to study the effect of sub-toxic level of oxidative stress. SB225002 (Sigma-Aldrich, Burlington, MA) of 0.3–1.0 µM was used for inhibition of CXCR2 in ARPE-19 cells. The primary antibodies used for western blot analysis, α-SMA, FN, and GAPDH, were purchased from Abcam (Cambridge, UK), whereas E-cad, Snail, and Slug were purchased from Cell Signaling Technology (Danvers, MA).

### mRNA expression analysis using real-time PCR

RNA extraction from ARPE-19 cells and real-time PCR were done following the protocol in the previous study with modification^43^. Briefly, total RNA was extracted from the cells by using the Qiagen RNeasy Mini Kit (Venlo, Netherlands). After quantification, 1 µg of total RNA was used to synthesize cDNA by reverse transcription with the oligo(dT)15 primer, M-MLV reverse transcriptase, and dNTP mixture with the procedure provided by manufacturer’s (Promega, Madison, WI). The expression of mRNA of target genes in ARPE-19 cells was detected by real-time quantitative PCR (RT-qPCR) using SYBR green dye with Light-Cycler Real-Time PCR system (Roche, Penzburg, Germany). The sequences of primers used in RT-qPCR are listed in Table 1.

**Table 1:** Primer list for real-time PCR.

Gene	Forward primer	Reverse primer
E-cadherin	CAC GGT AAC CGA TCA GAA TG	ACC TCC ATC ACA GAG GTT CC
α-SMA	CCG ACC GAA TGC AGA AGG A	ACA GAG TAT TTG CGC TCC GAA
Fibronectin	AAA CTT GCA TCT GGA GGC AAA CCC	AGC TCT GAT CAG CAT GGA CCA CTT
Snail	GGT CGT AGG GCT GCT GGA A	ACC ACT ATG CCG CGC TCT T
Slug	GAC CCT GGT TGC TTC AAG GA	TGT TGC AGT GAG GGC AAG AA
CXCR1	TGG GAA ATG ACA CAG CAA AA	AGT GTA CGC AGG GTG AAT CC
CXCR2	TTG TTG GCT CTT CTT CAG GG	TGA GGC TTG GAA TGT GAC TG
CXCL1	CCC AAG AAC ATC CAA AGT GTG	GTC ACT GTT CAG CAT CTT TTC G
CXCL2	GGG CAG AAA GCT TGT CTC AA	GCT TCC TCC TTC CTT CTG GT
CXCL3	CGC CCA AAC CGA AGT CAT AG	GCT CCC CTT GTT CAG TAT CTT TT
CXCL5	GAG AGC TGC GTT GCG TTT G	TTT CCT TGT TTC CAC CGT CCA
CXCL6	AGA GCT GCG TTG CAC TTG TT	GCA GTT TAC CAA TCG TTT TGG GG
CXCL8	AAG CTG GCC GTG GCT CTC TTG	AGC CCT CTT CAA AAA CTT CTC

### The enzyme-linked immunosorbent assay (ELISA)

The effect to H_2_O_2_ on the CXCL1 protein level in culture medium of ARPE-19 cell was assayed with ELSIA kit (abcam 190805) according to the procedure recommended by manufacture. Briefly, cells were seeded in a sterile transparent 96-well plate and cultured until fully confluent. The cells were then treated with H_2_O_2_ for 4 h then replaced by fresh medium for 20 h. After that, the plate was centrifuged and the supernatants were collected and incubated with mixture of capture and detection antibodies for CXCL1. The wells with samples were then washed to remove the unbound proteins and reacted with 3,3′,5,5′-tetramethylbenzidine development solution. The enzyme reaction was ended by adding stop solution. Absorbance at 450 nm was detected with Hidex Sense Multimodal Microplate Reader (Turku, Finland).

### Western blot analysis

The details of protein extraction from cultured cells and western blot analysis were reported before^43^. Briefly, whole-cell lysates of ARPE-19 were obtained by washing cells in 6.0-mm dishes with cold Dulbecco’s PBS (DPBS) and lysing them using mammalian protein extraction buffer (GE Healthcare, Little Chalfont, UK) with a protease inhibitor (Sigma-Aldrich). Cells were disrupted by sonication and supernatants were collected after pelleting cellular debris by centrifugation (10,000 × *g* at 4 °C for 10 min). Proteins were prepared using sample buffer containing 2-mercaptoethanol (2-ME), resolved by sodium dodecyl sulfate polyacrylamide gel electrophoresis (SDS-PAGE), and transferred to polyvinylidene difluoride (PVDF) membranes (EMD Millipore, Billerica, MA) that were blocked with SuperBlock blocking buffer (Thermo Scientific). Blots were probed with primary antibodies against target proteins overnight at 4 °C, and then incubated with horseradish peroxidase-linked secondary antibodies (EMD Millipore). Images were captured using the VersaDoc MP5000 enhanced chemiluminescence (ECL) imaging system (Bio-Rad, Hercules, CA).

### Cell viability assay

Cells were seeded in 96 well plates (5 × 10^4^ cells each well). After 80% confluency and replacement to serum free medium for 4 h, the cells were treated with H_2_O_2_ for 4 h then replaced by fresh medium for 20 h or TGF-β2 for 24 h. After individual treatment, the culture media was replaced with fresh Phenol red free media 10% (v/v) of CCK-8 reagent (Dojindo, Kumamoto, Japan) and incubated for 2 h at 37 °C in an incubator. The absorption of the medium was then measured at 450 nm with Hidex Sense Multimodal Microplate Reader (Turku, Finland).

### Statistical analysis

Data are presented either as mean ± standard deviation or as percentage. One-way ANOVA was employed for comparing the differences between groups in RT-qPCR and ELISA, and the Kruskal Wallis test was employed for analyzing results of western blot analysis with SPSS 13.0 software. P-value < 0.05 was considered statistically significant.

## Supplementary information

Supplementary Figures.

## References

[1] Kalluri R, Neilson EG (2003). Epithelial-mesenchymal transition and its implications for fibrosis. J. Clin. Invest..

[2] Kalluri R (2009). EMT: when epithelial cells decide to become mesenchymal-like cells. J. Clin. Invest..

[3] Kalluri R, Weinberg RA (2009). The basics of epithelial-mesenchymal transition. J. Clin. Invest..

[4] Cheresh P, Kim SJ, Tulasiram S, Kamp DW (2013). Oxidative stress and pulmonary fibrosis. Biochim. Biophys. Acta.

[5] Mastruzzo C, Crimi N, Vancheri C (2002). Role of oxidative stress in pulmonary fibrosis. Monaldi Arch. Chest Dis..

[6] Kinnula VL, Fattman CL, Tan RJ, Oury TD (2005). Oxidative stress in pulmonary fibrosis: a possible role for redox modulatory therapy. Am. J. Respir. Crit. Care Med..

[7] Poli G (2000). Pathogenesis of liver fibrosis: role of oxidative stress. Mol. Asp. Med..

[8] Zacks DN, Han Y, Zeng Y, Swaroop A (2006). Activation of signaling pathways and stress-response genes in an experimental model of retinal detachment. Invest. Ophthalmol. Vis. Sci..

[9] Jain M (2013). Mitochondrial reactive oxygen species regulate transforming growth factor-beta signaling. J. Biol. Chem..

[10] Beatty S, Koh H, Phil M, Henson D, Boulton M (2000). The role of oxidative stress in the pathogenesis of age-related macular degeneration. Surv. Ophthalmol..

[11] Liang FQ, Godley BF (2003). Oxidative stress-induced mitochondrial DNA damage in human retinal pigment epithelial cells: a possible mechanism for RPE aging and age-related macular degeneration. Exp. Eye Res..

[12] Bhatt L, Groeger G, McDermott K, Cotter TG (2010). Rod and cone photoreceptor cells produce ROS in response to stress in a live retinal explant system. Mol. Vis..

[13] Inumaru J (2009). Molecular mechanisms regulating dissociation of cell-cell junction of epithelial cells by oxidative stress. Genes. Cell.

[14] Takahashi E (2010). Tumor necrosis factor-alpha regulates transforming growth factor-beta-dependent epithelial-mesenchymal transition by promoting hyaluronan-CD44-moesin interaction. J. Biol. Chem..

[15] Maehata Y (2010). Reactive oxygen species (ROS) reduce the expression of BRAK/CXCL14 in human head and neck squamous cell carcinoma cells. Free. Radic. Res..

[16] Fernando RI, Castillo MD, Litzinger M, Hamilton DH, Palena C (2011). IL-8 signaling plays a critical role in the epithelial-mesenchymal transition of human carcinoma cells. Cancer Res..

[17] Liang C-M (2011). Glucosamine inhibits epithelial-to-mesenchymal transition and migration of retinal pigment epithelium cells in culture and morphologic changes in a mouse model of proliferative vitreoretinopathy. Acta Ophthalmologica.

[18] Agoulnik IU (2014). The Complex Interplay between ERK1/2, TGFβ/Smad, and Jagged/Notch Signaling Pathways in the Regulation of Epithelial-Mesenchymal Transition in Retinal Pigment Epithelium Cells. Plos One.

[19] Shephard P (2004). Myofibroblast differentiation is induced in keratinocyte-fibroblast co-cultures and is antagonistically regulated by endogenous transforming growth factor-beta and interleukin-1. Am. J. Pathol..

[20] Gallucci RM, Lee EG, Tomasek JJ (2006). IL-6 modulates alpha-smooth muscle actin expression in dermal fibroblasts from IL-6-deficient mice. J. Invest. Dermatol..

[21] Kuo PL, Shen KH, Hung SH, Hsu YL (2012). CXCL1/GROalpha increases cell migration and invasion of prostate cancer by decreasing fibulin-1 expression through NF-kappaB/HDAC1 epigenetic regulation. Carcinogenesis.

[22] Cheng WL (2011). Overexpression of CXCL1 and its receptor CXCR2 promote tumor invasion in gastric cancer. Ann. Oncol..

[23] Symeonidis C (2014). Chemokine CXCL-1 expression in the subretinal fluid during rhegmatogenous retinal detachment. Ocul. Immunol. Inflamm..

[24] Symeonidis C (2015). Chemokine CXCL-1: activity in the vitreous during proliferative vitreoretinopathy. Clin. Exp. Immunol..

[25] Cichon MA, Radisky DC (2014). ROS-induced epithelial-mesenchymal transition in mammary epithelial cells is mediated by NF-kB-dependent activation of Snail. Oncotarget.

[26] Lee SY (2019). Reactive oxygen species induce epithelialmesenchymal transition, glycolytic switch, and mitochondrial repression through the Dlx2/Snail signaling pathways in MCF7 cells. Mol. Med. Rep..

[27] Shen XH (2013). Interleukin-8 prevents oxidative stress-induced human endothelial cell senescence via telomerase activation. Int. Immunopharmacol..

[28] Guichard C (2005). Interleukin-8-induced priming of neutrophil oxidative burst requires sequential recruitment of NADPH oxidase components into lipid rafts. J. Biol. Chem..

[29] Wang L (2016). Genetic and Pharmacologic Inhibition of the Chemokine Receptor CXCR2 Prevents Experimental Hypertension and Vascular Dysfunction. Circulation.

[30] Ryu JK, Cho T, Choi HB, Jantaratnotai N, McLarnon JG (2015). Pharmacological antagonism of interleukin-8 receptor CXCR2 inhibits inflammatory reactivity and is neuroprotective in an animal model of Alzheimer’s disease. J. Neuroinflammation.

[31] Zhou SL (2015). CXCR2/CXCL5 axis contributes to epithelial-mesenchymal transition of HCC cells through activating PI3K/Akt/GSK-3beta/Snail signaling. Cancer Lett..

[32] Cherng S, Young J, Ma H (2008). Alpha-Smooth Muscle Actin (α-SMA). J. Am. Sci..

[33] Kawasaki Y (2008). Renal expression of alpha-smooth muscle actin and c-Met in children with Henoch-Schonlein purpura nephritis. Pediatr. Nephrol..

[34] Hinz B, Celetta G, Tomasek JJ, Gabbiani G, Chaponnier C (2001). Alpha-smooth muscle actin expression upregulates fibroblast contractile activity. Mol. Biol. Cell.

[35] Lamouille S, Xu J, Derynck R (2014). Molecular mechanisms of epithelial-mesenchymal transition. Nat. Rev. Mol. Cell Biol..

[36] To WS, Midwood KS (2011). Plasma and cellular fibronectin: distinct and independent functions during tissue repair. Fibrogenesis Tissue Repair..

[37] Clark RA, An JQ, Greiling D, Khan A, Schwarzbauer JE (2003). Fibroblast migration on fibronectin requires three distinct functional domains. J. Invest. Dermatol..

[38] Gui L, Wojciechowski K, Gildner CD, Nedelkovska H, Hocking DC (2006). Identification of the heparin-binding determinants within fibronectin repeat III1: role in cell spreading and growth. J. Biol. Chem..

[39] Zhou X (2008). Fibronectin fibrillogenesis regulates three-dimensional neovessel formation. Genes. Dev..

[40] Basson CT (1990). Spatiotemporal segregation of endothelial cell integrin and nonintegrin extracellular matrix-binding proteins during adhesion events. J. Cell Biol..

[41] Hinz B (2007). The myofibroblast: one function, multiple origins. Am. J. Pathol..

[42] Angst BD, Marcozzi C, Magee AI (2001). The cadherin superfamily: diversity in form and function. J. Cell Sci..

[43] Lee JJ (2012). High-mobility group box 1 protein is implicated in advanced glycation end products-induced vascular endothelial growth factor A production in the rat retinal ganglion cell line RGC-5. Mol. Vis..

