# Supplementary material for: Oxidative stress enhanced the transforming growth factor-β2-induced epithelial-mesenchymal transition through chemokine ligand 1 on ARPE-19 cell
Source: Sci Rep. 2020 Mar 4;10:4000. doi: 10.1038/s41598-020-60785-x (PMC7055234; doi:10.1038/s41598-020-60785-x)
Supplement: Supplementary file 1 — Supplementary Figures. [file 41598_2020_60785_MOESM1_ESM.pdf]

Supplementary Information

Title

Oxidative stress enhanced the transforming growth factor-β2-induced epithelial-mesenchymal transition through chemokine ligand 1 on ARPE-19 cell.

Authors

I-Hui Yang, Jong-Jer Lee, Pei-Chang Wu, Hsi-Kung Kuo, Yu-Hsia Kuo, Hsiu-Mei Huang*

Supplementary Fig. S1

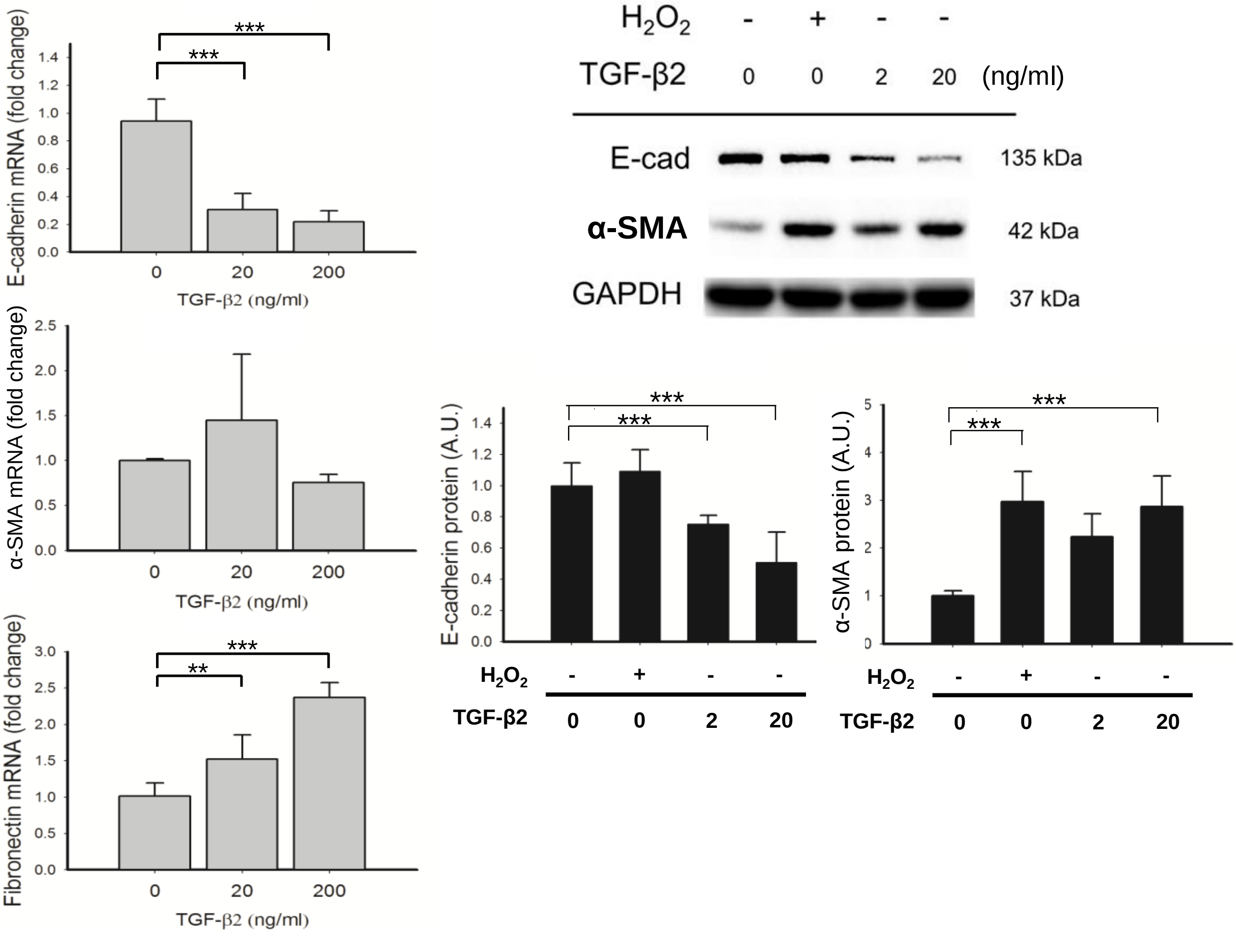

Supplementary Fig. S1. TGF-β2 induced EMT in APRE-19 cells. The mRNA of E-cad and FN were changed significantly after TGF-β2 20 ng/mL and 200 ng/mL. The protein expression of E-cad was suppressed but α-SMA was enhanced by TGF-β2 20 ng/mL. (Results of 4 repeated experiments; * $p < 0.05$; ** $p < 0.01$; *** $p < 0.001$).

Supplementary Fig. S2

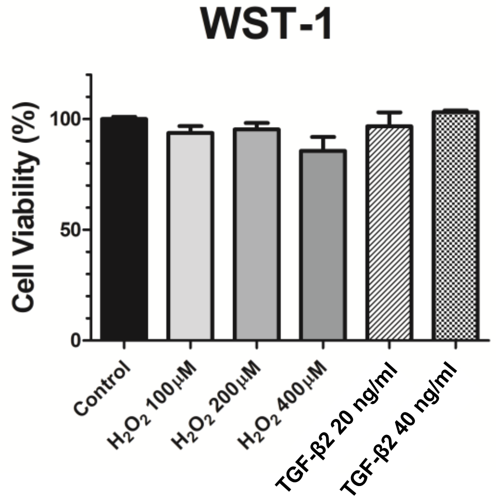

Supplementary Fig. S2. Treatment of hydrogen peroxide for 4h at a concentration below 400 µM or TGF-β2 of 20 to 40 ng/mL for 24 h did not change the cell viability when compared with PBS-treated ARPE-19 control cells. (Results of 4 repeated experiments).

Supplementary Fig. S3

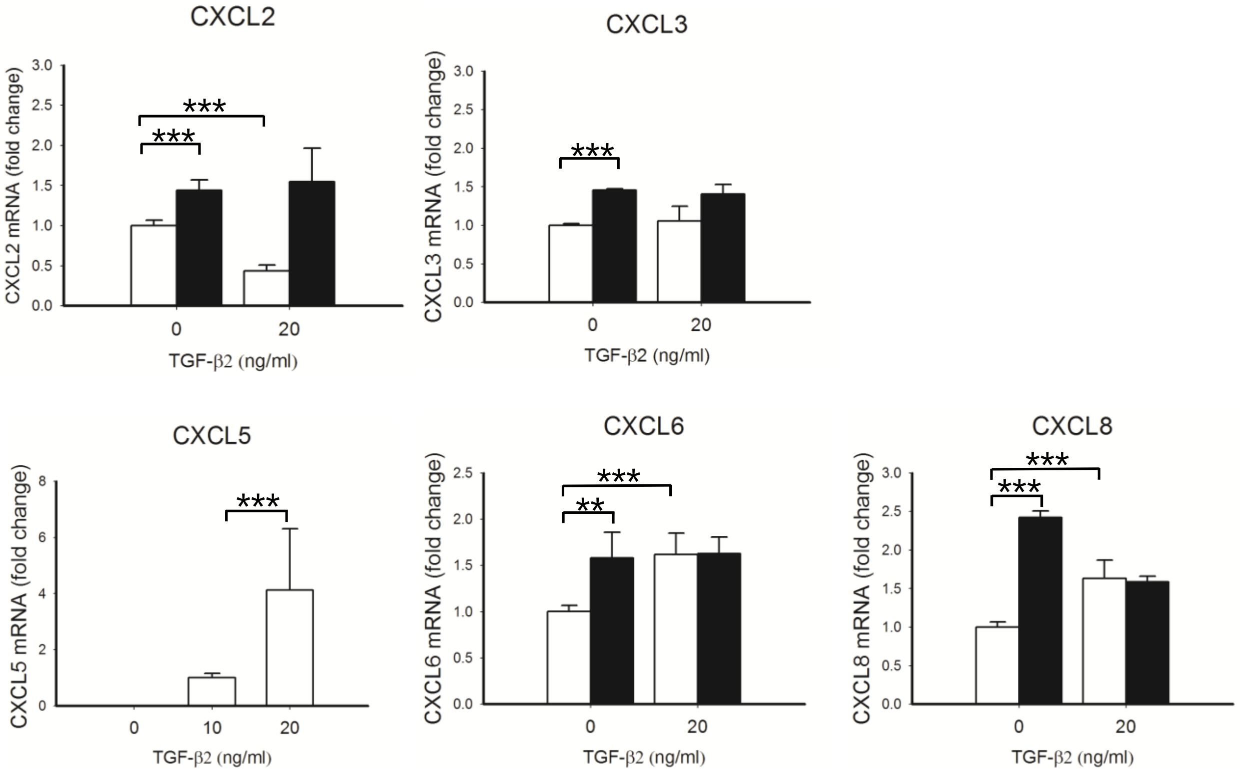

Supplementary Fig. S3. H_2O_2 and TGF-β2 showed different effect on mRNA expression of CXCL2, CXCL3, CXCL5, CXCL6 and CXCL8. (Results of 4 repeated experiments; * p < 0.05; ** p < 0.01; *** p < 0.001).
